# Full-endoscopic spine surgery for intradural pathologies: A systematic review of innovations, challenges, and future prospects

**DOI:** 10.1016/j.bas.2025.104392

**Published:** 2025-08-13

**Authors:** Ilyas Dolas, Tugrul Cem Unal, Cafer Ikbal Gulsever, Duygu Dolen, Ali Guven Yorukoglu, Altay Sencer

**Affiliations:** a Department of Neurosurgery, Istanbul Faculty of Medicine, Istanbul University, Istanbul, Turkey; b Department of Neurosurgery, Hakkari State Hospital, Hakkari, Turkey; c Department of Neurosurgery, Istanbul Scoliosis and Spine Center, Florence Nightingale Hospital, Istanbul, Turkey

**Keywords:** Full-endoscopic spine surgery, Intradural extramedullary tumors, Intradural pathologies, Minimally invasive neurosurgery, Spinal arachnoid cysts, Tethered cord syndrome

## Abstract

**Introduction:**

Full-endoscopic spinal surgery (monoportal endoscopy) has emerged as a minimally invasive alternative for managing intradural spinal pathologies, offering reduced morbidity and accelerated recovery compared to conventional techniques.

**Research question:**

What is the current evidence on the effectiveness, limitations, and future prospects of full-endoscopic spinal surgery for intradural pathologies?

**Material and methods:**

A systematic review following PRISMA guidelines was conducted. Electronic databases (Cochrane, OVID-MEDLINE, PubMed, Embase, Web of Science, Scopus) were searched for studies published from 2000 to 2024. Studies on full-endoscopic intradural spine surgery, including clinical trials, comparative studies, case reports, and meta-analyses in English, were included. Exclusion criteria involved non-intradural pathologies, biportal techniques, animal studies, and non-English publications. Narrative synthesis was performed due to study heterogeneity.

**Results:**

Thirty studies were reviewed. Full-endoscopic techniques effectively managed conditions such as tethered cord syndrome, spinal arachnoid cysts, intradural extramedullary tumors, Chiari malformation, and select vascular and inflammatory conditions. Benefits included minimal tissue disruption, improved visualization, shorter hospital stays, and reduced complications. Key challenges identified were intraoperative bleeding control, reliable dural closure, and irrigation-related complications like increased intracranial pressure.

**Discussion and conclusion:**

Full-endoscopic spine surgery demonstrates substantial clinical promise for intradural spinal conditions, significantly reducing morbidity and enhancing recovery. However, addressing current technical challenges is essential. Ongoing technological advancements in imaging, neuronavigation, and robotic-assisted systems, combined with future clinical trials, will be critical for expanding indications and confirming long-term efficacy.

## Highlights

• Full-endoscopic surgery treats complex intradural spine conditions.
• Minimally invasive approach reduces morbidity, speeds recovery.
• Challenges remain in bleeding control and dural closure methods.
• Innovative imaging and robotics promise better surgical outcomes.
• Further trials are needed to validate long-term clinical efficacy.

## 1 Introduction

Endoscopic techniques in spinal and cranial surgery have undergone substantial advancements over the past three decades. Among these, full-endoscopic spinal surgery, also known as monoportal or uniportal endoscopy, has emerged as a minimally invasive alternative, featuring a single working channel. Notably, the Yeung Endoscopic Spine System (YESS) and Ruetten's fully endoscopic interlaminar technique have revolutionized lumbar discectomies by enabling safer and more efficient access to the epidural space while preserving critical anatomical structures (Yeung, 1999; Ruetten and Komp, 2020).

While biportal endoscopic techniques, utilizing two separate channels, have been increasingly employed for complex spinal procedures such as thoracic intradural extramedullary tumor resections, full-endoscopic approaches remain distinctive due to their inherently minimal tissue disruption and superior visualization capabilities (Tredway et al., 2007; Dhandapani and Karthigeyan, 2018). The single-channel configuration significantly reduces the operative footprint, enhancing surgical precision, minimizing morbidity, and accelerating patient recovery (Dhandapani and Karthigeyan, 2018; Di, 2009).

The historical application of endoscopic visualization of intradural spinal structures dates back as early as 1931. However, significant technological and methodological advances in the late 20th and early 21st centuries have facilitated the evolution of full-endoscopic methods from purely diagnostic tools to viable surgical alternatives for a variety of intradural pathologies (Chern et al., 2011; Endo and Tominaga, 2020). Conditions traditionally managed through open or microscopic surgery, such as tethered cord syndrome, spinal arachnoid cysts, and intradural extramedullary tumors, are now increasingly treated using these minimally invasive endoscopic approaches, offering notable reductions in postoperative morbidity and accelerated rehabilitation timelines (Di, 2009; Endo and Tominaga, 2020; Magrassi et al., 2008).

This systematic review aims to comprehensively examine the historical progression, current clinical applications, existing limitations, and prospective developments in full-endoscopic spine surgery for intradural pathologies, underscoring its expanding role as a standalone surgical approach within contemporary neurosurgical practice.

## 2 Methods

### 2.1 Study design and guidelines

This systematic review was conducted according to the Preferred Reporting Items for Systematic Reviews and Meta-Analyses (PRISMA) guidelines (Page et al., 2021). A comprehensive literature search was performed using electronic databases, including Cochrane, OVID-MEDLINE, PubMed, Embase, Web of Science, and Scopus, to identify studies published from January 2000 to December 2024. The keywords used were: “full-endoscopic spine surgery,” “monoportal endoscopy,” “total endoscopic,” “pure endoscopic,” “intradural pathologies,” “tethered cord syndrome,” “spinal arachnoid cysts,” “intradural extramedullary tumors,” “functional neurosurgery,” and “CSF leak.” The search was limited exclusively to English-language articles.

### 2.2 Inclusion and exclusion criteria

Studies eligible for inclusion were clinical trials, case reports, comparative studies, and meta-analyses explicitly addressing full-endoscopic (monoportal) techniques for intradural spinal surgeries. Comparative studies that evaluated full-endoscopic methods against other surgical approaches were included if they provided relevant outcome data, such as perioperative morbidity, complication rates, and patient recovery times.

Studies were explicitly excluded if they primarily focused on biportal endoscopic techniques or other minimally invasive spinal approaches unrelated to monoportal endoscopy, addressed extradural pathologies, involved animal models, were review articles without original comparative data, or were published in languages other than English. This explicit focus on monoportal techniques ensured clarity and specificity in assessing the effectiveness and challenges of full-endoscopic surgery for intradural spinal conditions.

### 2.3 Study selection

**Fig. 1:**
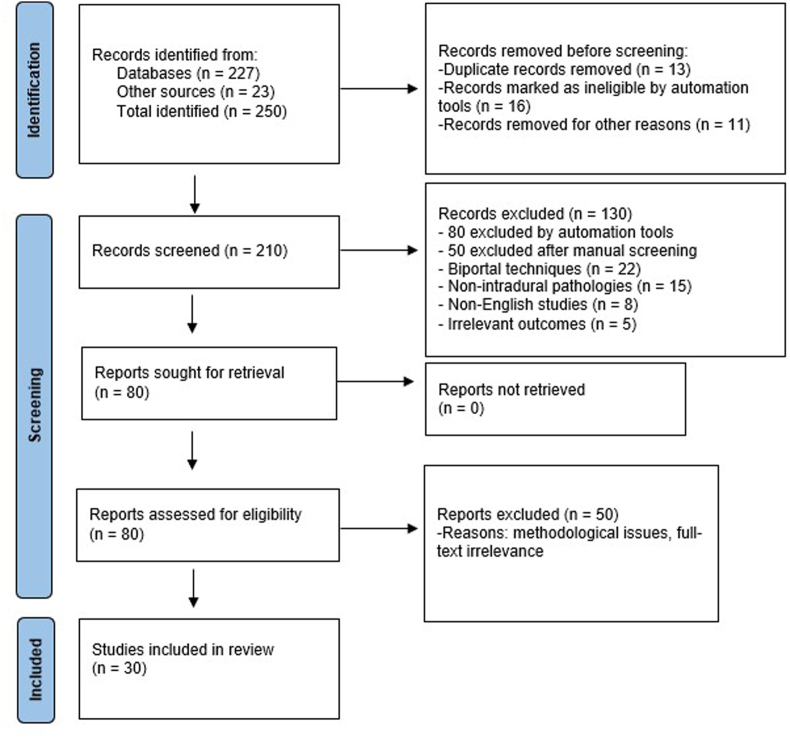
PRISMA flow diagram illustrating the study selection process for the systematic review of full-endoscopic spine surgery for intradural pathologies. A total of 250 records were identified from databases and other sources. After removing duplicates, automated exclusions, and other irrelevant records, 210 records were screened for eligibility. After screening, 80 full-text articles were sought for retrieval and assessed for eligibility. A total of 50 reports were excluded based on methodological issues or irrelevance upon full-text review. Finally, 30 studies were included in the qualitative synthesis.

The study selection process was depicted using a PRISMA flow diagram. Initially, 250 records were identified (227 from databases and 23 from additional sources), from which 40 were excluded due to duplication or irrelevance before screening. Of the remaining 210 records screened based on titles and abstracts, 130 studies were excluded. Subsequently, detailed assessment of 80 full-text articles resulted in the exclusion of 50 studies due to their focus on biportal techniques (n = 22), irrelevant pathologies (n = 11), non-intradural focus (n = 12), animal studies (n = 2), or publication in non-English languages (n = 3). Ultimately, 30 studies met all criteria and were included in the qualitative synthesis (Fig. 1).

### 2.4 Data extraction and synthesis

Data were systematically extracted from each included study, focusing on the type of intradural pathology, surgical techniques used, clinical outcomes (perioperative morbidity, complication rates, and recovery time), and duration of follow-up. Given significant heterogeneity among studies regarding design, outcomes measured, and patient populations, a narrative synthesis was performed instead of a formal meta-analysis.

## 3 Results

This systematic review identified significant advancements in the application of full-endoscopic spine surgery techniques for various intradural pathologies. A total of 30 studies were included (Table 1), highlighting the effectiveness, advantages, challenges, and limitations associated with these minimally invasive methods. Key findings are summarized according to specific intradural conditions.

**Table 1:** Summary of studies on full-endoscopic surgery for intradural spinal pathologies. **Abbreviations:** AV, arteriovenous; CNS, central nervous system; IDEM, intradural extramedullary; N/A, not applicable; SAC, spinal arachnoid cyst; TCS, tethered cord syndrome.

	Author, Year	Study Design	Sample Size	Intradural Pathology	Surgical Technique	Key Findings	Complications	Follow-up
**1**	Tani et al., 2003 (Tani et al., 2003)	Case reports	2	Anterior Sacral Meningocele	Endoscopic surgical strategy	Effective management of an anterior meningocele	None reported	5 months
**2**	Oertel and Burkhardt, 2017 (Oertel and Burkhardt, 2017)	Case series	9	CSF Leak Management	Endoscopic dural tear treatment (Muscle, fascial graft, tissue sealant)	Effective dural closure methods	None reported	12 weeks
**3**	Arishima et al., 2017 (Arishima et al., 2017)	Technical note	N/A	CSF leak with superficial siderosis	Endoscopy combined with CT myelography	Precise localization and dural closure	None reported	N/A
**4**	Shin et al., 2018 (Shin et al., 2018)	Technical note	N/A	CSF Leak Management	Youn's endoscopic suturing	Effective innovative dural repair	None reported	N/A
**5**	Ratre et al., 2018 (Ratre et al., 2018)	Case series	15	Chiari Malformation	Endoscopic decompression (Outer dural layer opening only)	Effective decompression	Two patients had occasional headaches, and four patients had sensory disturbances	9–21 months
**6**	Dolas et al., 2023 (Dolas et al., 2023a)	Cadaveric study	5	Chiari Malformation	Full-endoscopic decompression (With dural opening)	Effective decompression	None reported	N/A
**7**	Dolas et al., 2023 (Dolas et al., 2023b)	Case report	2	Chiari Malformation	Full-endoscopic decompression (Without dural opening)	Effective decompression	None reported	6 months
**8**	Staribacher et al., 2023 (Staribacher et al., 2023)	Case report	1	Chiari Malformation	Full-endoscopic decompression (Dura mater opened, closed by dural substitute patch)	Effective decompression	None reported	N/A
**9**	Barami and Dagnew, 2007 (Barami and Dagnew, 2007)	Case report	2	IDEM tumors (Meningioma, primary meningeal melanocytic tumor)	Endoscope-assisted posterior resection	Successful resection of ventral IDEM tumors	None reported	N/A
**10**	Zhu et al., 2015 (Zhu et al., 2015)	Case series	18	IDEM tumors (Schwannoma, ependymoma, epidermoid cyst, enterogenous cyst)	Dura closed by titanium dural clips	Effective dural closure, tumor removal	One patient had urinary incontinence	3–12 months
**11**	Parihar et al., 2017 (Parihar et al., 2017)	Case series	18	IDEM tumors (Schwannoma, meningioma)	Endoscopic resection, endoscopic dural stay sutures	Effective minimally invasive removal	None reported	9–24 months
**12**	Senturk and Unsal, 2019 (Şentürk and Üü, 2019)	Case report	1	IDEM tumors (Meningioma)	Endoscopic resection, closure with tissue glue	Minimally invasive, effective tumor removal	None reported	3 months
**13**	Ahn, 2020 (Ahn, 2024)	Case report	1	IDEM tumor (Schwannoma)	Full-endoscopic resection	Effective minimally invasive removal	None reported	N/A
**14**	Zhang et al., 2023 (Zhang et al., 2023)	Case series	5	IDEM tumors (Schwannoma, meningioma)	Endoscopic resection, closure with artificial dura	Effective IDEM tumor removal	None reported	6–12 months
**15**	Hu et al., 2023 (Hu et al., 2023)	Case series	25	IDEM tumor (Schwannoma)	Endoscopic resection (Interlaminar, extraforaminal)	Effective IDEM tumor removal	One patient had transient voiding difficulty	3 months–6 years
**16**	Hagel and Van Isseldyk, 2024 (Hagel and Van Isseldyk, 2024)	Case report	1	IDEM tumor (Schwannoma)	Endoscopic resection, closure with inlay Gelfoam and outlay dural patch application	Effective IDEM tumor removal	None reported	16 month
**17**	Fukushima et al., 2018 (Fukushima et al., 2006)	Case report	1	Inflammatory Disease (Neurosarcoidosis)	Spinal endoscopic biopsy	Effective minimally invasive diagnosis of CNS neurosarcoidosis	None reported	N/A
**18**	Torres-Corzo et al., 2019 (Torres-Corzo et al., 2019)	Case report	3	Neurocysticercosis	Flexible neuroendoscopy removal	Safe cysticerci removal	None reported	2–5 years
**19**	Fonoff et al., 2010 (Fonoff et al., 2010)	Technical note	1	Neuropathic Pain	Percutaneous radiofrequency cordotomy	Effective pain management	Ipsilateral Horner sign, and mild upper limb ataxia	6 months
**20**	Tanaka et al., 1997 (Tanaka et al., 1997)	Case report	1	Spinal Arachnoid Cysts	Endoscopic treatment of spinal subarachnoid cysts	Effective symptomatic relief of spinal subarachnoid cysts	None reported	15 months
**21**	Mauer et al., 2008 (Mauer et al., 2011)	Clinical study	28	Spinal Arachnoid Cysts	Arachnoscopy (Spinal intradural endoscopy)	Specialized endoscopic technique effective for arachnoid cysts	Increased syrinx size, reoperation	1–5 years
**22**	Endo et al., 2010 (Endo et al., 2010)	Case series	6	Spinal Arachnoid Cysts	Endoscopic cyst fenestration	Effective cyst fenestration, no recurrence	None reported	52–163 months
**23**	Tan et al., 2019 (Tan et al., 2019)	Case report	1	Spinal Arachnoid Cysts	Endoscopic cysto-subarachnoid shunt placement	Successful arachnoid adhesiolysis	Sensory loss in the right lower limb	2 months
**24**	Magrassi et al., 2008 (Magrassi et al., 2008)	Case report	1	Tethered Cord Syndrome	Total endoscopic cauda approach	Effective minimally invasive approach	None reported	12 months
**25**	Di, 2009 (Di, 2009)	Case report	2	Tethered Cord Syndrome	Full-endoscopic tethered cord release	Effective minimally invasive approach	None reported	3 months, 2 years
**26**	Yörükoglu et al., 2016 (Yörükoǧlu et al., 2016)	Cadaveric study	18	Tethered Cord Syndrome	Percutaneous endoscopic sectioning	Reduced morbidity, minimal trauma	None reported	N/A
**27**	Telfeian et al., 2017 (Telfeian et al., 2017)	Case report	1	Tethered Cord Syndrome	Minimally invasive endoscopic spinal cord untethering	Successful untethering, minimal morbidity	None reported	12 months
**28**	Endo et al., 2014 (Endo et al., 2014)	Case series	8	Spinal vascular malformation (arteriovenous shunts)	Endoscopic approach for cervical perimedullary AV shunts	Effective management emphasizing angioarchitecture	Gait disturbance, mild motor weakness	6–90 months
**29**	Ito et al., 2017 (Ito et al., 2017)	Case report	1	Spinal vascular malformation (AV fistulas)	Indocyanine Green Fluorescence Endoscopy	Successful treatment of concurrent perimedullary and dural AVFs	None reported	3 years
**30**	Mansour et al., 2019 (Mansour et al., 2019)	Case report	1	Spinal vascular malformation (aneurysm)	Endoscopic fluorescence-guided aneurysm clipping	Successful aneurysm clipping at the craniocervical junction	None reported	2 years

### 3.1 Tethered cord syndrome (TCS)

Full-endoscopic interlaminar approaches demonstrate particular promise in managing tethered cord syndrome (TCS), especially in cases associated with fatty filum terminale. Traditionally, TCS required open or microscopic surgical intervention; however, recent advancements highlight the efficacy of minimally invasive endoscopic techniques. Initial endoscopic attempts primarily focused on diagnostic visualization rather than direct surgical intervention. Early studies utilized intrathecal endoscopy primarily to enhance diagnostic accuracy, improving the visualization of tethered structures (Woods et al., 2010). A landmark cadaveric and subsequent clinical study by Yörükoğlu et al. demonstrated successful full-endoscopic percutaneous sectioning of the filum terminale, significantly minimizing tissue disruption and postoperative morbidity, thereby confirming clinical feasibility and safety (Yörükoǧlu et al., 2016). Furthermore, case reports by Di and Telfeian et al. supported these findings, emphasizing the success of minimally invasive untethering with reduced morbidity and rapid recovery (Di, 2009; Telfeian et al., 2017). Additionally, full-endoscopic techniques have been successfully adapted for related pathologies, such as anterior sacral meningocele (ASM) and split cord syndrome, facilitating simultaneous filum sectioning and cyst ligation with minimal morbidity (Tani et al., 2003).

### 3.2 Spinal arachnoid cysts (SACs)

Full-endoscopic surgery has proven highly effective in managing symptomatic spinal arachnoid cysts (SACs), especially those spanning multiple spinal segments. Traditional surgical interventions typically required extensive multi-level laminectomies, significantly increasing postoperative morbidity and the risk of spinal instability or deformities. In contrast, full-endoscopic techniques substantially reduce invasiveness by utilizing rigid and flexible endoscopes, enabling precise cyst fenestration or shunt placement with minimal bone removal, thus preserving spinal stability.

Several studies have demonstrated these advantages. Early reports, such as those by Tanaka et al., demonstrated effective symptomatic relief of spinal subarachnoid cysts using endoscopic methods, with no notable complications noted over a 15-month follow-up period (Tanaka et al., 1997). Additionally, a clinical series by Mauer et al. described “arachnoscopy,” a specialized form of spinal intradural endoscopy, as highly effective for treating arachnoid cysts. However, they reported reoperation due to increased syrinx size in some patients over a 1- to 5-year follow-up period (Mauer et al., 2011). Endo et al. further supported these findings, reporting effective cyst fenestration with no recurrence observed over an extended follow-up period (52–163 months) (Endo et al., 2010). More recent individual case reports, such as that by Tan et al., also documented successful cysto-subarachnoid shunt placement with minimal morbidity, despite temporary sensory deficits in one case (Tan et al., 2019).

### 3.3 Intradural extramedullary lesions (IDEM)

Intradural extramedullary (IDEM) tumors, such as schwannomas, meningiomas, and metastases, traditionally treated via open or microscopic surgery, have increasingly benefited from full-endoscopic resection techniques. Since the initial reports, including a notable early study by Barami et al., which successfully employed endoscope-assisted posterior resections of ventral IDEM tumors, there has been significant advancement in endoscopic techniques aimed at minimizing operative trauma and enhancing patient recovery (Barami and Dagnew, 2007).

Recent literature consistently demonstrates the effectiveness of full-endoscopic methods. Zhu et al. described successful endoscopic resection of IDEM tumors, including schwannomas, ependymomas, and epidermoid cysts, achieving effective dural closure using titanium clips (Zhu et al., 2015). Parihar et al. further reported a series of 18 patients undergoing fully endoscopic resection of schwannomas and meningiomas, emphasizing minimal invasiveness, lower complication rates, and shorter hospital stays compared to conventional microsurgical approaches (Parihar et al., 2017). Similarly, Senturk and Unsal documented successful percutaneous endoscopic removal of a meningioma, further reinforcing the feasibility and advantages of these techniques (Şentürk and Üü, 2019).

Recent larger series provide additional support. Hu et al. reported the outcomes of full-endoscopic resection in 25 schwannoma cases, achieving excellent clinical results and minimal complications over an extended follow-up period ranging from 3 months to 6 years (Hu et al., 2023). Hagel et al. confirmed these results, demonstrating the effectiveness of full-endoscopic schwannoma resection without recurrence at 16-month follow-up (Hagel and Van Isseldyk, 2024). Additionally, Zhang et al. reported the successful removal of IDEM tumors with artificial dura application for closure in five patients, further enhancing safety and effectiveness (Zhang et al., 2023). Yong Ahn, emphasized the clinical significance and technical feasibility of the monoportal full-endoscopic technique, highlighting its value in reducing surgical morbidity and enhancing patient recovery in complex intradural cases (Ahn, 2024).

### 3.4 Spinal vascular malformations

Although less commonly reported, full-endoscopic approaches for spinal vascular malformations have shown significant promise, especially for managing intricate lesions such as aneurysms, arteriovenous fistulas (AVFs), and arteriovenous (AV) shunts. Early pioneering studies, notably by Endo and Tominaga, demonstrated effective endoscopic clipping of anterior spinal artery aneurysms, emphasizing enhanced visualization of critical vascular and neural anatomy through angled endoscopes (Endo and Tominaga, 2020). Similarly, Mansour et al. utilized endoscopic fluorescence imaging to successfully clip an anterior spinal artery aneurysm at the craniocervical junction, underscoring the potential for fluorescence-guided techniques to improve surgical safety and accuracy (Mansour et al., 2019).

Recent case series have further validated these findings. Endo et al. described successful endoscopic treatment of cervical perimedullary AV shunts in a cohort of eight patients, achieving effective lesion management by clearly delineating complex angioarchitecture and significantly reducing operative morbidity (Endo et al., 2014). Additionally, Ito et al. successfully treated concurrent perimedullary and dural AVFs using Indocyanine Green (ICG) fluorescence endoscopy, providing enhanced intraoperative visualization and enabling precise intervention without complications (Ito et al., 2017).

### 3.5 Intramedullary lesions and inflammatory diseases

Intramedullary lesions, including tumors and inflammatory conditions, present significant surgical challenges due to their critical location within the spinal cord. Nevertheless, full-endoscopic techniques have demonstrated significant potential, particularly for minimally invasive diagnostic biopsies and management of inflammatory diseases such as neurosarcoidosis. Fukushima et al. described a successful spinal endoscopic biopsy for diagnosing central nervous system neurosarcoidosis, highlighting the minimal invasiveness, diagnostic accuracy, and safety of the procedure (Fukushima et al., 2006).

Additionally, spinal endoscopy has proven beneficial in managing parasitic infections like spinal neurocysticercosis. Torres-Corzo et al. demonstrated the utility of flexible neuroendoscopy in effectively visualizing and removing cysticerci, thereby restoring cerebrospinal fluid (CSF) flow without damaging spinal cord tissues, and further highlighting the versatility and potential clinical applications of spinal endoscopic techniques beyond conventional surgical indications (Torres-Corzo et al., 2019).

### 3.6 Chiari malformation

Full-endoscopic methods have been successfully applied to posterior fossa decompression in Chiari I malformation. Minimally invasive endoscopic techniques, utilizing small incisions and tubular retractors, provide superior visualization compared to traditional open approaches. Early clinical experience by Ratre et al., reporting 15 patients undergoing endoscopic decompression with outer dural-layer opening, demonstrated effective decompression, although minor complications such as transient headaches and sensory disturbances occurred (Ratre et al., 2018).

Recent anatomical and clinical studies by Dolas et al. further advanced these techniques. Dolas et al. conducted an anatomical feasibility study on five human cadavers, demonstrating successful posterior fossa decompression through a full-endoscopic technique, highlighting its safety and feasibility (Dolas et al., 2023a). Subsequently, Dolas et al. applied the same technique clinically to two patients without dural opening, confirming its clinical effectiveness and minimal invasiveness at a 6-month follow-up (Dolas et al., 2023b). In addition, Staribacher et al. successfully performed a full-endoscopic decompression involving dural opening and repair using a substitute patch, underscoring ongoing improvements in dural closure methods (Staribacher et al., 2023).

### 3.7 Functional neurosurgery and chronic pain management

Full-endoscopic techniques have demonstrated significant potential for innovative applications in functional neurosurgery, particularly in managing chronic pain syndromes such as neuropathic pain and spasticity unresponsive to conventional therapies. The advent of small-caliber endoscopes has facilitated precise interventions, including minimally invasive percutaneous radiofrequency cordotomy and selective rhizotomies. Fonoff et al. reported successful outcomes with percutaneous endoscopic-guided radiofrequency cordotomy, effectively relieving neuropathic pain while minimizing collateral tissue damage. Although minor, transient side effects such as Horner's syndrome and mild upper limb ataxia occurred, the overall morbidity was significantly reduced compared to traditional open techniques (Fonoff et al., 2010, 2011).

Critically, it must be emphasized that the core advantage of endoscopic assistance in intradural pain management interventions, unlike superficial procedures such as facet denervation, is improved visualization and precision when accessing deeper neural structures, which traditional fluoroscopy-based percutaneous techniques do not provide. While general anesthesia is sometimes necessary, potentially increasing invasiveness compared to superficial percutaneous procedures, the enhanced visualization offered by endoscopy may substantially improve procedural accuracy, safety, and clinical outcomes, thus justifying its targeted use in selected, more complex intradural pain management procedures rather than routine superficial pain interventions.

### 3.8 Cerebrospinal fluid (CSF) leak management

Cerebrospinal fluid (CSF) leaks remain a common complication in spinal surgery, especially in intradural procedures. Endoscopic approaches have proven effective both for identifying and repairing dural defects, often facilitated by adjunctive imaging methods such as CT myelography. Arishima et al. successfully employed spinal endoscopy combined with CT myelography, achieving precise localization and effective closure of spinal dural defects associated with superficial siderosis (Arishima et al., 2017). Various innovative dural closure techniques have further enhanced endoscopic outcomes. Oertel and Burkhardt demonstrated successful dural repair methods employing muscle, fascial grafts, and tissue sealants (Oertel and Burkhardt, 2017). Additionally, Shin et al. introduced a specialized endoscopic suturing technique ("Youn's technique”) providing robust and reliable dural closure in endoscopic lumbar surgeries (Shin et al., 2018). Despite these advancements, achieving consistently reliable dural sealing remains challenging, highlighting the ongoing need for technological refinement in endoscopic instruments and techniques.

## 4 Discussion

The application of full-endoscopic spine surgery for intradural pathologies has demonstrated significant promise, supported by growing evidence of reduced perioperative morbidity, shorter recovery times, and enhanced surgical precision compared to traditional open and microscopic techniques (Di, 2009; Endo and Tominaga, 2020; Yörükoǧlu et al., 2016). In this systematic review, we provide a detailed, data-driven synthesis of existing evidence regarding these techniques. By systematically evaluating 30 carefully selected studies, we extracted and summarized scientific data, including study designs, sample sizes, follow-up durations, surgical techniques, clinical outcomes, and complication rates. Nevertheless, this review also emphasizes the existing challenges and technical limitations that currently constrain the widespread adoption of full-endoscopic methods (Oertel and Burkhardt, 2017; Kang et al.). This comprehensive analysis allows for a critical and evidence-based evaluation of full-endoscopic approaches, substantiating conclusions regarding their effectiveness, safety, limitations, and future potential.

### 4.1 Advantages and clinical implications

Substantial clinical evidence supports the efficacy of full-endoscopic methods across several intradural conditions (Mauer et al., 2011; Endo et al., 2010, 2014; Tan et al., 2019; Hu et al., 2023; Hagel and Van Isseldyk, 2024; Zhang et al., 2023; Mansour et al., 2019; Ito et al., 2017). For tethered cord syndrome (TCS), multiple studies demonstrated significantly reduced operative trauma and accelerated patient recovery compared to traditional open surgery. Yörükoğlu et al., for instance, demonstrated in cadaveric and clinical models the feasibility and safety of percutaneous full-endoscopic filum terminale sectioning with minimal morbidity (Yörükoǧlu et al., 2016). Similarly, Telfeian et al. and Di independently corroborated excellent outcomes with minimally invasive endoscopic spinal cord untethering, further supporting endoscopy's clinical advantages in TCS management (Di, 2009; Telfeian et al., 2017).

For spinal arachnoid cysts (SACs), extensive series and case reports collectively confirm sustained symptomatic relief and reduced complications with full-endoscopic approaches. Endo et al. reported excellent clinical outcomes in a case series of six patients, demonstrating no recurrence over long-term follow-up periods (52–163 months) (Endo et al., 2010). Further supporting this, Mauer et al. showed the effectiveness of specialized arachnoscopy for spinal arachnoid cysts, though some reoperations were required due to syrinx enlargement, highlighting technical considerations and limitations (Mauer et al., 2011).

In the management of intradural extramedullary (IDEM) tumors, robust clinical data supports full-endoscopic methods as equal or superior to microsurgical techniques in select cases. Zhu et al. and Parihar et al. demonstrated successful IDEM tumor removal, highlighting reduced operative trauma, shorter hospital stays, and fewer complications compared to conventional approaches (Zhu et al., 2015; Parihar et al., 2017). Recent larger series further confirmed these benefits; Hu et al. reported successful gross total resections in 24 out of 25 cases, with minimal complications and no recurrence during a median follow-up of 22 months, substantiating endoscopy's efficacy and safety in tumor management (Hu et al., 2023).

### 4.2 Insights from comparative minimally invasive techniques

While full-endoscopic approaches have distinct benefits, comparisons with biportal endoscopic and other minimally invasive techniques offer valuable insights. Dhandapani and Karthigeyan, comparing microendoscopic (biportal) and pure endoscopic (monoportal) approaches, observed that biportal techniques provide enhanced bimanual control and facilitate easier dural repair due to their dual-channel configuration. However, biportal methods inherently require larger incisions, more extensive muscle dissection, and frequently use tubular retractors, thus somewhat diminishing their minimally invasive advantages relative to monoportal full-endoscopy (Dhandapani and Karthigeyan, 2018; Zhu et al., 2015).

Similarly, Zhu et al. demonstrated successful removal of intradural extramedullary (IDEM) lesions utilizing microendoscopic methods, emphasizing superior dural closure capabilities due to the availability of bimanual manipulation. Nevertheless, such biportal techniques necessitate broader surgical access and more extensive soft tissue disruption compared to the strictly single-channel monoportal full-endoscopic procedures (Zhu et al., 2015).

While biportal endoscopy remains beneficial for complex intradural pathologies requiring extensive suturing or meticulous dural repairs, monoportal full-endoscopic methods uniquely minimize soft tissue trauma, accelerate patient recovery, and enhance cosmetic outcomes. However, to fully capitalize on these advantages, technical challenges such as bleeding control and dural closure must be adequately addressed (Dhandapani and Karthigeyan, 2018; Zhu et al., 2015).

### 4.3 Challenges and technical limitations

Despite its considerable advantages, full-endoscopic spinal surgery encounters several notable challenges. Hemostasis remains a primary concern due to the limited working space, complicating effective bleeding control within narrow endoscopic channels. Kang et al. emphasized the critical need for precise hemostatic strategies during endoscopic spinal procedures, highlighting the potential risk of converting to open surgery if bleeding control is inadequate (Kang et al.).

Another significant technical limitation is achieving consistently reliable dural closure using monoportal techniques. Innovative solutions such as titanium dural clips, fibrin glue, and specialized endoscopic suturing methods like Youn's technique have been demonstrated by Oertel and Burkhardt and Shin et al. (Oertel and Burkhardt, 2017; Shin et al., 2018). Nonetheless, achieving a robust and watertight dural closure remains particularly challenging, especially in the context of larger or irregularly shaped dural defects (Oertel and Burkhardt, 2017; Shin et al., 2018).

Elevated intracranial pressure (ICP) resulting from prolonged irrigation represents an additional critical limitation. This complication was notably illustrated by Wu and Fang, who reported postoperative seizures attributed to increased ICP caused by excessive irrigation fluid accumulation during endoscopic lumbar surgery. Consequently, careful optimization of irrigation systems and diligent intraoperative monitoring are essential to mitigate these associated risks (Wu et al., 2020).

### 4.4 Future directions and technological innovations

Future advancements in full-endoscopic spinal surgery are expected to address current technical limitations significantly. Enhanced imaging modalities, robotic assistance, and flexible endoscopes integrated with advanced neuronavigation systems may substantially improve precision and procedural safety (Li and Wang, 2021; Carl et al., 2019). Emerging technologies such as augmented reality (AR) and intraoperative magnetic resonance imaging (MRI) hold promise for enhancing intraoperative visualization, enabling real-time assessment, and potentially reducing complications related to inadequate surgical exposure or incomplete lesion resection (Carl et al., 2019; Liounakos and Wang, 2020; Jitpakdee et al., 2023).

Moreover, further comparative studies examining full-endoscopic versus biportal techniques are needed to establish clear indications and optimal surgical approaches for specific spinal pathologies. Robust, multicenter randomized controlled trials will be essential in validating long-term clinical outcomes, safety profiles, and cost-effectiveness of full-endoscopic spinal surgery methods (Li and Wang, 2021; Jitpakdee et al., 2023).

### 4.5 Limitations of the review

This systematic review has several limitations. The studies included varied significantly regarding design, population size, outcome measures, and follow-up periods, making quantitative meta-analysis infeasible. The narrative synthesis approach adopted herein provides an overview but lacks the statistical power of a meta-analysis. Additionally, restricting the search to English-language publications might have introduced publication bias, potentially excluding valuable data from non-English sources. Finally, by excluding studies explicitly focused on biportal or other minimally invasive techniques except for comparative insights, we may have inadvertently limited the contextual understanding of alternative endoscopic methods, although we have addressed key comparative findings explicitly within the discussion.

## 5 Conclusion

Full-endoscopic spine surgery represents a highly promising, minimally invasive approach for managing intradural spinal pathologies, including tethered cord syndrome, spinal arachnoid cysts, intradural extramedullary tumors, Chiari malformations, and selected vascular and inflammatory conditions. This systematic review, synthesizing outcomes from 30 rigorously selected studies, confirms substantial reductions in morbidity, shorter recovery periods, and fewer complications compared to traditional surgical methods. However, critical challenges remain regarding bleeding control, reliable dural closure, and irrigation-related complications. Continued technological innovation, combined with robust prospective clinical studies and standardized outcome assessments, are necessary to fully overcome these limitations and firmly establish full-endoscopic techniques as a standard of care for intradural spinal surgery.

## Author contributions

Conceptualization: Ilyas Dolas, Cafer Ikbal Gulsever.

Formal Analysis: Ilyas Dolas, Tugrul Cem Unal, Altay Sencer.

Investigation: Cafer Ikbal Gulsever, Duygu Dolen.

Methodology: Ilyas Dolas, Tugrul Cem Unal, Cafer Ikbal Gulsever, Ali Guven Yorukoglu.

Project Administration: Duygu Dolen, Ali Guven Yorukoglu, Altay Sencer.

Writing – Original Draft: Ilyas Dolas, Cafer Ikbal Gulsever, Ali Guven Yorukoglu.

Writing – Review & Editing: Ilyas Dolas, Ali Guven Yorukoglu, Altay Sencer.

## Declaration of competing interest

The authors declare that they have no conflicts of interest concerning the publication of this article. This research did not receive any specific grant from funding agencies in the public, commercial, or not-for-profit sectors.
